# Abstracts and Gleanings

**Published:** 1885-10-20

**Authors:** 


### ABSTRACTS AND GLEANINGS.

**Hemorrhagic Malarial Fever.**—The following cases are extracted from a paper by Dr. I. J. Newton, Jr., in the Transactions of the Louisiana State Medical Society :

CASE I.

G. H——, age twenty-six years, states that he has had chills occasionally for six months prior to his present illness ; that several times during the past ten years has had several severe malarial attacks of sickness, but never of the hæmaturic form ; enlargement of the spleen has existed, he thinks, for three or four months.

On November 24, 1884, had a chill, followed by high fever. At his own suggestion, 10 grains of quinine was taken in three doses to prevent recurrence of chill.

On the 21st, at 8 o'clock a. m., had a very severe chill, followed by high fever ; incessant vomiting ; sudden supervention of jaundice ; urine bloody and voided frequently, copious in quantity, lessening, however, in quantity and time as fever subsided, and at 6 o'clock p. m. his urine was normal in appearance, and jaundice very slight. Medical aid was sent for but was not procured ; a large dose of sulphate of magnesia was taken at 10 o'clock p. m., which acted on bowels.

On the 22d patient had a recurrence of above symptoms, the nausea and vomiting perhaps more intense, his urine of darker color. The chill occurred about 8 a. m. I saw him for the first time at 2 o'clock p. m. ; found symptoms as above stated ; an hour later his fever was subsiding and with it the attendant symptoms enumerated. Examination of the urine passed early in the attack, appeared almost black and yielded a play of bile colors on addition of nitric acid ; from the deposits thrown down I believed blood also to be present in considerable quantity. Urine voided at time I first saw him is of much lighter color, but contains both bile and blood. At 6 p. m. urine normal in appearance.

*Diagnosis.*—Hemorrhagic malarial fever of intermittent type.

*Treatment*—Immediately, after seeing patient, he was ordered a hot mustard foot-bath, and $\frac{1}{8}$ gr. mur. pilocarpin was administered hypodermically, which induced a mild diaphoresis ; 15 grains of calomel, in three portions, one to be taken every three hours, and followed, if necessary, by a seidlitz powder ; 20 grains of quinine in ten powders, one powder to be given every hour, beginning at 10 p. m. This prevented the return of the paroxyism.

I then ordered :

℞. Acid arseniosi............................gr. j,
Quiniæ sulph...............................gr. xxx,
Ferri redact...............................gr. xx,
Podophylin................................ gr. ij,
Ol. pip nigr. ..............................gtt. x.

M. et ft. pil. xx.

Sig : One pill three times daily, at meals.

### Case II.

On September 7, 1881, I was called in consultation to see H——, age seventeen, living in a swampy locality, and who had been suffering from repeated attacks of intermittent malarial fever. On the day before he was seized with a severe chill, lasting for two hours, followed by violent fever and jaundice, and bloody urine, these passing away, however, on subsidence of fever. He was ordered by the attending physician a saline cathartic and 12 grains of quinine in three portions, the last portion to be given four hours before time for paroxysm to return. Notwithstanding this, at about the same time on the 8th, chill returned, followed by symptoms same as yesterday, but more intense.

I saw him five hours after chill had occurred. At this time he presented the following symptoms: Intense jaundice; hot and dry skin; pulse 140; temperature in axilla 105½°; nausea incessant, vomiting dark green bilious matter; delirious, but easily aroused; intellect clear; a constant desire to get over the urinal, and voids urine copiously, loaded with bile and blood. Believing that in this case the kidneys were the organs threatened with rapid structural changes, and knowing the utter uselessness at this stage to depend upon either quinine or calomel, we had the patient placed over a vessel of hot water, covered him with a blanket, and placed iced cloths to his head; he was allowed to receive the vapor bath until his skin began to perspire freely, when he was wiped thoroughly dry, given hypodermically ¼ gr. sul. morphia and 1-120 gr. sul. atropia, and replaced in bed. He slept several hours, and on awakening voided urine almost normal in appearance, and wsa, in every respect, very much better. Appropriate doses of quinine and calomel completed the cure.

### Case III.

This patient is a married lady, thirty-seven years of age, of stout frame, and has not been sick from any cause for several years; other members of the family, however, had suffered from intermittent malarial fever for several months prior to this lady's attack.

On Monday, September —, she was quite well; Tuesday, ailing.

On Wednesday had a decided chill, followed by fever lasting five or six hours.

On Thursday another chill, more severe than the former, causing her to remain in bed after subsidence of fever. No medicines were taken, except a seidlitz powder on Tuesday.

On Friday, chill returned more severe than ever, followed, as stated by patient, by a not very high fever. Immediately following the chill she became intensely jaundiced; nausea and vomiting persistent; urine copious and of black appearance. Dr. B—— was called at this stage of the attack, and immediately began the administration of quinine in large and repeated doses, which were, however, not retained. No other medicines were given. The patient did not complain of any pain whatever, and seemed disposed to stupor. The chill came on at 5 o'clock a. m.; at 8 a. m. she was seen by Dr. B. At 1 o'clock p. m. she was in profound stupor, and could with great difficulty be aroused, when she would answer intelligently, stating she was free from pain, but very, very sleepy. At this time I first saw the patient. With considerable difficulty she was aroused, and with continual slapping with cold wet towels she was kept aroused sufficiently long to void a large quantity of urine thoroughly charged with bile and possibly some blood. The appearance of the urine in urinal was intensely black, stains yellow, and gave a beautiful play of bile colors on addition of nitric acid ; the sputa was yellow ; perspiration stains were also yellow, and serum, under a blister drawn over the liver, was yellowish. In fact, her whole system seemed *deluged* with bile. Inquiry ascertained the fact that she was obstinately constipated, and had been so throughout the attack ; quiet when undisturbed, yet nausea and vomiting were easily excited, vomiting almost everything given her on stomach. Failing to unload bowels by stimulating enemas, and also failing to excite the skin to action, the patient was, at 2.30 p.m., given 30 grains of calomel, dry on tongue, washed down by a draught of iced water ; patient kept awake by constant interference, as slapping with wet towels, etc. At 3.30 another 30-grain dose of calomel was administered. An hour later an enema of warm soap water was administered, but without effect ; one hour later a repetition of a similar enema caused copious thick, tarry-looking motions, which were kept up at short intervals for eight or ten hours, the patient being relieved from the stupor, but remaining somewhat sleepy. Calomel in 5-grain doses was continued for twenty-four hours, at intervals of six hours.

The kidneys had never stopped their action, and in twenty-four hours after chill the urine was of normal appearance, skin only slightly jaundiced, and patient in every respect much improved.

Only a nourishing treatment was given after this, until convalescence was thoroughly established, when she was given an arsenic and iron preparation. Convalescence was slow, and months elapsed before her former health was regained.

Instances of this disease in types as above stated being very rare, I have deemed it advisable to give you this imperfect history of these cases. Hoping to have made my views clear and intelligible, I now submit this paper for your consideration.

DISCUSSION.

Dr. R. H. Day said he saw his first case of hemorrhagic malarial fever in the year 1837, whilst practicing at Mount Carmel, Ill.

Dr. Joseph Jones said cases of parasitic hemorrhagic fever were found in Africa and the Island of Mauritius ; that a number of cases which are classed as hemorrhagic are really melanuric. The point of special interest is to determine the cause of this fatal disease. He regards the cultivation of rice in Louisiana as very detrimental to the health of her inhabitants.

Dr. Newton asked Dr. Jones if cases of parasitic hemorrhagic fever are not of a mild type.

Dr. Jones replied in the negative.

Dr. O. P. Langworthy said that he agreed with Dr. Newton in his views about this fatal disease, although he differed somewhat as to treatment. He placed much faith in the use of small doses of calomel with large doses of acetate of potash.

Dr. T. B. Pugh asked if the treatment of this disease with quinine was good practice.

Dr. J. B. Gazzo said that malarial fever made its appearance in Latourche Parish about the first of September, and remained until about the first of December. He used quinine in its treatment, but he had found, when sulphuric acid was used to dissolve it, the blood became defibrinated; such was not the case, however, if citric acid was used.

Dr. Lyon said that he is satisfied that he has seen harm done by large doses of quinine.

Dr. Sutherlin stated that he gave nothing but quinine; that he administered 10 grains every two hours until he had given 60 grains, and in some cases he administered as much as 190 grains in twenty-four hours.

Dr. R. W. Seay, of East Carroll Parish, said only a few cases of this dise occurred in his parish. He related the following history of a case occurring in his practice: A gentleman complained, one evening, of feeling badly, and stated that he had just passed a large quantity of dark urine. The doctor gave him large doses of nitre and oil of copabia, and directed that he void his urine in a vessel so that he might examine it. In a few hours he passed about one and a half pints of very dark (black) urine, the specific gravity of which was 1028°. The patient had no fever nor pain; pulse fre quent. In the morning, the urine was clear, and the specific gravity had fallen to 1020°. Under the use of moderate doses of quiniae, nitre, oil of wintergreen, and oil of copaiba, for three days, recovery was complete, and there was no recurrence of the disease.

The doctor said that he had been informed by the physicians practicing at Greenville, Miss., that they used quinine in the treatment of this disease.

**When Quinine fails, What then?**—Dr. Matas (North Carolina Medical Journal) says, in reference to fevers resisting quinine: "It is now four years since I began the administration of sodium salicylate in fever practice as a succedaneum to quinine, whenever this salt failed to act antithermically, and since that time I have constantly resorted to it, often with astonishing success in all continued fever cases, and particularly in yellow fever.

"Since the introduction of kairin (by Fischer and Filchne) and antipyrin (by Knorr, Filchne, Pensoldt, and Sartorius) I have resorted to these drugs, and, though my experience with them has been limited to nine fever cases, six of which were of the long-continued type, I have met with such striking and uniform success in lowering hyperthermic fever, that I am almost prompted to state that these agents are the most potent antipyretics in the materia medica.

"I have observed several cases in which sodium salicylate had failed to produce an impression, and in which antipyrin repeatedly succeeded in bringing down very high temperatures to almost the normal degree.

"In my experience, which is confirmatory of that of most observers who have studied this drug, antipyrin is preferable to kairin, the latter drug often giving rise to gastric disturbances, nausea, vomiting, etc.

"Antipyrin, as a rule, is well borne, and lowers temperature more gradually and permanently than kairin. Its effects, in all my cases, have been far less disagreeable than quinine, the patients usually taking the drug very readily in capsules.

"In the treatment of fever in children, who are unable to swallow capsules, or to whom the administration of a liquid solution of antipyrin would be troublesome, the exhibition of this compound by enema is very efficacious, the rectum usually retaining the solution far better than quinine, which is often rejected on account of the irritant character of its acid solution.

"In cases in which there is great prostration, and particularly a weak pulse, the practitioner should be wary as to the dose in which he employs these antipyretics. Thus far all observers express a belief in the benignity of the physiological action of antipyrin, but the recent report of a death, supposed to be due to the free administration of antipyrin, should make us careful. I believe, however, that in doses of 5, 10, 15, and even 20 grains, it will never exercise any detrimental action.

"I have given it quite freely in some recent cases, and not the slightest depressing effect have I observed on the pulse.

"In three cases of long-continued fever which I have had under my charge since January, the medicinal (antithermic) plan of treatment adopted was as follows: In the beginning, when the diagnosis was uncertain, and simple malarial remittent was suspected, quinine was given largely and freely—20, 30 and 40 grains a day. When, after the thorough production of chinchonism, no effect was noticeable on the temperature, sodium salicylate was administered in the following manner:

"℞. Sodii salicylatis .................................ʒ ii,

Syr. papaveris .................................℥ i,

Syr. aurant. flor.................................℥ i,

Aquæ q. s. ad.................................℥ vi.

"S: one tablespoonful of this mixture every hour, whenever the temperature rise above 102°. If any cardiac prostration manifested itself by excessive frequency and irritability of pulse, 2 drachms of tinct. of digitalis were added to the solution.

"The reason for administering the salicylate only when the temperature is above 102° F., is based on the observation that sodium salicylate appears to lower only high temperatures. A fever of 104° or 105° is much more quickly diminished by the salicylate than would be a fever in which the thermometer registered only 102° or less.

"There are some cases in which the salicylate appears to lose its antithermic potency, and particularly when dealing with these long-continued fevers. In such cases antipyrin (if I am to judge by my recent experience) will rarely, if ever, fail in reducing the fever. I usually prescribe it in capsules, viz:

"℞. Antipyrin.................................gr. xxx.

"Ft. in caps. No. XV.

"S: Two capsules every half hour until ten are taken, remaining five at an hour's interval.

"The temperature will almost invariably fall *pari passu* with the administration of the drug.

"With these three agents, viz: sodium salicylate, kairin, antipyrin (and perhaps also the still more recent thallin, which I have not yet tried), the practitioner is fully and powerfully prepared to encounter excessive thermic movements.

"Of course, it should be well understood that, while claiming for these agents real and most efficient antithermic properties, I do not believe that they do in any manner abbreviate the course of a fever; they are not specific remedies, such as quinine is for the malarial poison—they are simply heat-regulators, and as such are agents of priceless value at certain periods in the career of these long-fever cases. I simply recommend them here as a basis for a *medicinal* antithermic therapensis in cases where quinine is impotent as an antipyretic.

"Whenever the practitioner is allowed to apply the cold bath I would apply the method which Dr. Guiteras has found so efficient in Key West. But I fear that the marked prejudice which exists in our population against the cold bath in fever would allow but a very limited and unsatisfactory application of the methods of Brande and of Leibermeister.

"Sponging of the surface with dilute *eau sedative* vinegar, etc., are not objected to in this locality, and consequently can be adopted with advantage to the patients.

"Finally, whilst advocating a vigilant and active antithermic treatment, I would not, in any manner, depreciate or underestimate the importance of constant alimentation, stimulation and all the various auxiliary measures comprised in good nursing. This is as important in the long tissue-wasting thermic fever of this section as in the more dreaded and devastating typhoid fever.

"Symptomatic remedies and other accessory measures of treatment are not mentioned, as they will readily suggest themselves to any educated practitioner whenever indications for their use present themselves."

**A Case of Cholera Infantum, Treated with Large Doses of Morphine and Atropia.**—Dr. G. B. Lawrason, in the New Orleans Med. Journal says: A child seven months old, was brought to me from Algiers, verging on a state of collapse. Its eyes were sinking, the face pinched, extremities cold, pulse thready and rapid; it was very much emaciated, and it would occasionally utter piteous and feeble whines as if in pain. The mother said that she had weaned the child about six weeks previously, and had since been feeding it on cow's milk and condensed milk. From that time, however, it had lost flesh steadily and its bowels had been out of order. First, the child had diarrhœa, then attacks of vomiting and purging, which, within the last two days, had become very frequent. It was always crying for the bottle and the parents had had no rest with the child for a week.

A powder composed of one-twentieth of a grain of morphine and one-sixtieth of a grain of atropia was placed dry upon the tongue, and, in a very short time, the child was as red as a lobster and sound asleep. In about two hours and a half it waked up, and, according to directions given at the consultation, ten drops of brandy in a teaspoonful of milk were given every halt hour while awake. The child was allowed the bottle, which, however, was to contain only water, whenever it wished.

I saw the father the next morning. He said the child was better and had only vomited once since the powder had been given, but that it still seemed very feeble. Three grains of musk in mucilage were ordered to be given by enema, and instructions repeated to keep up the brandy and milk as formerly directed. The next day I went to Algiers and found the child had made excellent progress towards recovery. It seemed to take notice and smile when amused, but suffered from occasional paroxysms of colic. Ten drops of paragoric were ordered every half hour until pain ceased. The brandy and milk were stopped and pancreatized milk substituted, a tablespoonful every half hour while awake. In addition to this, a nutrient enema, consisting of a teaspoonful of cod liver oil, emulsified with a little ether, with three teaspoonfuls of pancreatized milk, was given twice a day. The child recovered rapidly.

The pancreatized milk was made by dissolving a powder composed of five grains of pancreatic extract (Fairchild Bros. & Foster's) and ten grains of carbonate of soda, in a little warm water, and adding a pint of milk in a bottle. The bottle was then placed in hot water and allowed to remain twenty minutes. The milk was then poured out and boiled. If kept in a cool place, milk thus prepared will be fit for use all day. Should the milk be sour after going through this process, the child will refuse it. Such milk should be thrown away and new milk prepared in the same way, except that it should be left in the hot water a shorter time, say fifteen minutes. The morphine was suggested to me by an article in the American Journal of Obstetrics, in which large doses of morphine are recommended in cholera infantum to allay pain and procure rest. The atropia I added, thinking that it would ably second the morphine, diminish the risk and lessen the congestion of the alimentary tract. The dose of atropia may seem large, but it is my belief, based partly on the authority of Fothergill, and partly on my own experience, that babies resemble rabbits in their tolerance of this drug.—*N. O. Med. Journal.*

**Scutellaria Lateriflora in the Treatment of Enuresis.**—A writer in the N. Y. Medical Record says: "Some two years ago I was advised by an old practitioner to use fluid extract of scullcap in a case in my own family, and the result was most gratifying. I have since employed it in several cases dependent upon nervous conditions only, and must say that results obtained have always been most satisfactory. A lad, twelve years of age, who would urinate from three to six times every night during sleep, and had done so for several years, received 1 drachm t. i. d. for two weeks, and was speedily and permanently cured. All the cases occurred in children."

He advises a trial, at least, of scutellaria when other remedies have failed. He disclaims any credit for originality in the use of the drug, as he says it was recommended to him by another.

**Treatment for Malaria.**—Dr. I. R. N., writing to the Medical World, says: During my medical life I have resided in all kinds of malarial sections of our State, and have had considerable practice in what is generally known as malaria. With proper diagnosis, the different forms of it made no difference with regard to my treatment, which I began with the following:

℞. Mass. hydrarg............................gr. vj,
Ext. taraxici..............................gr. xviij,
Ipecac. pulv...............................gr. j.
M. et. ft Pill, No. vj.

Sig. One pill every four hours until all are taken, and if no motion of the bowels, order a saline.

Then I begin with the old, old remedy, quinia in solution, as follows:

℞ Quiniæ sulph.............................℈ j,

Acidi sulph. aromat........................q. s,

Capsici pulv...............................gr. iv,

Glycerini, aquæ aa.........................℥ j.

M. Dose: Tablespoonful every three hours.

Should this arrest the paroxysm, I then put my patient upon arsenic; if otherwise, I continue the quinine until the paroxysms are arrested. Then, to keep them off, I give arsenic, as follows:

℞. Fowler's solu...........................℥ j.

Dose: Five drops in a tablespoonful of water after eating, three times per day, until its physiological effects are noticed.

If I see that my patient needs a tonic, I order the following:

Take of columb, Virginia snake root, wild cherry bark, powdered gentian, each............................2 drachms.

Mix. Infuse by putting all into one pint of boiling water in an earthen crock, and simmer down to one-half pint, then strain, and add to the infusion one-half pint of good rye whisky.

Dose: A tablespoonful or two before meals.

I find it necessary in some cases to continue the quinine, 8 grains every six days for four weeks. I can say that I have had little difficulty in breaking up the chills and fever. I know that I have, in many instances, broken in upon a bilious or remittent fever by giving the hydrarg. et. tarax. pills.—*Medical Digest.*

**A Valuable Remedy for Headache.**—The Physician's and Surgeon's Investigator desires to call attention to a simple, and at the same time wonderfully efficient, treatment for many kinds of headache:

We lay no claims to originality, nor do we hnow who the originator was, but having used it for a year or more, and in many cases with remarkable results, we feel disposed to give it our endorsement, and desire to make it more generally known. The remedy is nothing more nor less than a solution of the bisulphide of carbon. A wide-mouth glass-stoppered bottle is half filled with cotton or fine sponge and upon this two or three drachms of the solution are poured. When occasion for its use occurs the mouth of the bottle is to be applied to the temple or as near as possible to the seat of pain, so closely that none of the volatile vapor may escape, and retained there four or five minutes, or longer For a minute or so nothing is felt, then comes a sense of tingling, which in a few minutes—three or four usually—becomes rather severe, but which subsides almost immediately if the bottle be removed, and any redness of the skin that may occur will also quickly subside. It may be re-applied, if necessary, several times in the day, and it generally acts like magic, giving immediate relief.

We believe this was the basis of a once popular nostrum. The class of headache to which it seems especially adapted is that which may be grouped under the broad term of "nervous." Thus neuralgic, periodic, and hysterical headaches are almost invariably relieved by it. True, the relief of a mere symptom is quite another thing from the removal of a cause, yet no one who has seen the distress, and even agony, caused by severe and frequently recurring headaches (and who has not ?) but will rejoice to be able to afford relief in so prompt and simple a manner ; besides, it is sure to secure the hearty gratitude of the patient if he has suffered long. As to the *modus operandi* we have nothing more definite than a theory to offer, and that is that the vapor being absorbed through the skin produces a sedative effect upon the superficial nerves of the part in which it is applied. We know by experience that its influence is not due to its power as a counter irritant. We however know that it does act, and if we do not clearly see in what way it acts, that is no more than can be said of several other remedies which are firmly established in professional favor and confidence.—*Weekly Medical Review.*

**Never Overlook an Over-distended Bladder.**—A writer in the Maryland Medical Journal reproduces from the British Med. Journal the histories of several cases of retention of urine, in which the over-distended bladder was taken for an abdominal tumor. In the comments following, a case is related in which the writer was called in consultation to examine a woman recently confined, in whom incontinence of urine had led to the suspicion of vesico-vaginal fistula. The withdrawal of three quarts of offensive urine cleared up the diagnosis.

The case last related recalls very vividly an incident in the lying-in ward of the Charity Hospital, which occurred some years ago, in the days of that great clinician, Prof. Frank Hawthorn. A two hundred-pound negro woman recently confined, was "passing her water in bed" to the satisfaction of the nurse and the resident student. But to the professor, on his morning visit, the words in quotation were ominous. A gum-elastic catheter was introduced in the presence of the medical class, and to the present writer more especially, it did seem that the flow of urine would never cease.

On the 28th of July, a negro was admitted into the Charity Hospital with retention of seven pints of highly colored and offensive urine. The paralysis of the bladder resulting has required catheterism up to the present writing, August 17th, and in a man of his advanced age, will result in permanent disability of the organ.—*N. O. Med. Journal.*

**Hydrobomate of Quinine and Valerinate of Caffeine in the Treatment of Malarial Poisoning.**—Cerededo ("Gazz. degli Ospit."; "Rev. Med.") concludes from numerous experiments that the hydrobromate of quinine is preferable to the sulphate for the following reasons:

1. Its activity is greater in moderate doses.
2. It acts as a nervous sedative.
3. It stops vomiting, a matter of special importance in certain countries.
4. It readily brings about a favorable change in the type of the fever.
5. Its bitterness is less marked.
6. It does not irritate the intestinal mucous membrane, and produces neither constipation nor diarrhœa.
7. It allows of the avoidance of too frequent subcutaneous injections.
8. It diminishes the probability of relapse.
9. When once the paroxysm has come on, if it cannot reduce its intensity and duration, it should be administered in capsules, combined with valerinanate of caffeine.
10. Given in that way half an hour before the paroxysm, it is capable of arresting the latter.
11. By combining the two salts (fifteen grains of the hydrobromate and seven or eight grains of the valerianate) we may check certain quotidian forms which are rebellious to much larger doses of sulphate.
12. The action of this combination of the two drugs, given in comparatively small doses, is more powerful than that of any other salt of quinine in much larger doses.—*N. Y. Med. Journal.*

**A Novel Cure for Rheumatism.**—El Siglo Medico relates the following singular cure from La Paz, Bolivia: A woman had suffered so much from rheumatism, that for six months she had hardly slept. Her right arm was so affected that it was quite useless; she could not work with it or dress herself. While in this state she heard of a countryman who suffered in the same way, and who had been cured by the accidental sting of a bee. As the pain caused by the sting could not be worse than that due to the rheumatism, she determined to try the same remedy. Three bees were obtained and made to sting her on the right arm. The success of the treatment was surprising and complete. On the following night she was able to sleep, and the acute pain had all but completely disappeared. The arm was naturally a good deal swollen, owing to the sting, but the swelling quickly disappeared with cold water dressings. The use of the arm gradually returned, and since there has been no symptom of rheumatism. It is said that the same remedy has been equally successful in several other persons.—*Medical and Surgical Reporter.*

**Local Anæsthetics.**—The facility of producing local anæsthesia has always been a great need in surgery. The most successful of the earlier attempts in this direction, the freezing spray, was attended with several inconveniences, and although the use of nitrous oxide and rapid respiration offered very satisfactory substitutes for the greater anæsthetics in short operations, yet they were still open to objection. The enthusiasm which prevailed a few months ago, when the striking properties of cocaine were announced. was evidence that it filled a widespread want. It is gratifying to know that extended experience has not seriously impaired the estimate which was first placed on its merits, but that its properties as a local anæsthetic are well marked and uniform. The field, however, is such that we can easily receive additions, and it is interesting to note that very lately two new agents, similar to cocaine, have been added. In a recent number of the Therapeutic Gazette, Dr. Thos. J. Mays, of Philadelphia, has detailed experiments which show that the alkaloid brucia has distinct local anæsthetic powers. This observation is interesting from two points of view: It is gratifying to know that so important a property is possessed by a body which is in abundant supply ; secondly, it throws light upon the toxicology of brucia. This alkaloid has always been regarded as analogous to strychnia, but of much less activity. Several cases of brucia poisoning are detailed in the standard works on toxicology, symptoms analogous to those of strychnia being presented. Bearing in mind the fact that the methods of separating proximate principles were originally imperfect, we can see that commercial brucia was little more than impure strychnia. Dr. Mays has pointed out that the only absolutely pure brucia will give the local anæsthetic effects, and that the commercial article gives the physiological reaction of strychnia. It is evident, therefore, that the toxicology of brucia will have to be revised. The co-relation between physiological investigation and chemical analysis is neatly demonstrated by these observations.—*Polyclinic.*

**Morphine in Cholera Infantum.**—Struck by the analogy between cholera infantum and cholera morbus, it occurred to Dr. Spencer M. Free (American Journal of Clinics, July, 1885) to use the same principle of treatment ; and since most authors advocate the use of morphine subcutaneously in the last named disease, in full doses, he was led to employ the same treatment in three cases of undoubted cholera infantum. In each of these cases the condition appears to have been extremely serious, and in all a turn for the better was inaugurated by the injections of morphine. As regard to the dose which he employs: In one case $\frac{1}{8}$ of a grain of morphine, combined with 1-300 of a grain of atrophine, was employed, and in the other two cases the morphine was given internally, in the form of powder, in doses of 1-12 of a grain. The after-treatment consisted, in the first place, in extreme care in diet, allowing, at first, nothing but milk and lime-water, with a mixture containing subnitrate of bismuth, wine of pepsin, tincture of opium, chalk mixture, and simple syrup. The particular advantage of this plan of treatment seems to be that perfect rest is secured from vomiting, diarrhœa, and other bad symptoms, and Nature is then afforded an opportunity to recover her balance, and to properly receive and apply our assistance. Of course, as regards the dose in this, as in all other diseases, each case must be a law unto itself, and the dose given must be regulated by the various attending circumstances and conditions. Dr. Free insists, however, that the dose must be large enough to produce its full physiological effect, or, in other words, cessation of all bad symptoms and perfect rest for several hours. The morphine should be given hypodermically or dry on the tongue. It should not be given in mixture, as it will then be most likely be rejected by the stomach. Absorption should occur through the mouth or subcutaneous tissues, and thus relieve the alimentary tract.—*Therapeutic Gazette.*

**Coincident Variola and Vaccinia.**—C. E. Rowling reports in the Australasian Medical Gazette, of February 15, 1885, pp. 114–15, a series of very important facts bearing on the relationships of variola to vaccinia. A man aged twenty-seven, presented himself to Dr. Rowling, stating that he was living in a house where there had been three cases of small-pox; and, as he was unvaccinated, he solicited advice. Dr. Rowling vaccinated the man immediately, in four places on each arm. The following day the man had pains in his back, and 102.6° F. of fever. Next day the temperature rose to 103°, and variolous eruption was declared, forty-three umbilicated pustules appearing on the body—three on the face, eleven on the chest, seventeen on the back and the rest on the legs. In due time the vaccinia took also its course. The vaccination was successful in every place, eight well-developed Jennerian vesicles appearing, four on each arm. The small-pox ran a very mild course. The case so far is full of interest, but the most important part has to be related. Dr. Rowling, with two other medical friends, vaccinated a child eight months old, from the clear lymph of one of the Jennerian vesicles on the arm of the variolous vaccinia patient, the result being that nothing but a normal attack of vaccinia occurred. They then vaccinated thirteen others, eleven of which were successful, two negative.

Dr. Rowling considers that this experience affords proof direct that vaccination carefully performed yields no result except vaccinia; and it would certainly be difficult to possess any evidence more thoroughly crucial.—*Polyclinic.*

**An Improvement in the Treatment of Nævus by Sodium Ethylate.**—In the treatment of nævus by sodium ethylate much time may be lost in waiting for the removal of the crust which is formed after the ethylate has been applied. While waiting for the loosening of the crust it often happens that the nævus undergoes fresh growth beneath, exhibiting a very red and irritable surface after the crust is removed for reapplication of the ethylate. In order to prevent these difficulties I have modified the plan of operation in a way which, in two cases, has proved most successful. The modification is as follows :

Over the nævus, at the first application I apply the ethylate very freely, adding a second layer of it when all the watery oozing has ceased. This causes in three days an extremely firm crust. On the third day, while the crust is still firm, instead of removing it, I puncture, through it in three or more places, with a sharply-edged, small flat needle, and with the needle I break up the vascular surface beneath freely. On withdrawing the needle a few drops of blood exude from the needle points, which blood I take up with cotton wool, pressing firmly so as to empty the surface entirely of blood. Then I apply over the surface a little ethylate, and do no more for four days. At the end of that time, if on pressing the crust there is evidence of fluid underneath it, I once more insert the needle and treat as before, but if the scale be flat and firm, I let it remain until it falls off of itself and leaves an even and healthy surface.—*The Asclepiad.*

**The Uses of Cocaine.**—Decidedly there is a future for cocaine. It is destined to have a permanent place in medicine, surgery, and dentistry. The scope of its uses is not yet defined, but it is safe to say that its applications are widening as experiments with it are extended. We have been especially impressed with this fact in looking over the literature of the subject recently issued by the house of Parke, Davis & Co., Detroit. They have published several most interesting pamphlets; one is entitled "Cocaine in Dental Surgery," another is a working bulletin on the drug, containing a variety of original material, and a third, a well composed collation of what has been reported about it in home and foreign medical literature. These pamphlets will be sent without charge by the house to any one requesting them.

The same firm has devised a very handy and ingenious "Cocaine Case," which they sell at a moderate price, and which impresses us as the best of the kind we have seen.—*Phil. Med. and Surg. Reporter.*

**Extirpation of the Lung**—It is said that "Dr. Domenico Biondi, of Naples, some time since, proved that animals recovered after removal, by operation, of one entire lung. In a more recent communication, in the Weiner Medizinische Jabrbucher, the same physician shows that animals may survive the removal of portions of lung artificially infected with tubercle. After injecting, by Ehrlich's method, masses of baccillus tuberculosis into the parenchyma of the lung, so that the clinical and anatomical symptoms of tubercle were produced, he removed, at the end of a few weeks, the diseased lungs ; and in all cases recovery was complete."

